# Temperate grassland songbird species accumulate incrementally along a gradient of primary productivity

**DOI:** 10.1371/journal.pone.0186809

**Published:** 2017-10-23

**Authors:** William L. Harrower, Diane S. Srivastava, Cindy McCallum, Lauchlan H. Fraser, Roy Turkington

**Affiliations:** 1 Department of Forest and Conservation Sciences, University of British Columbia, Vancouver, British Columbia, Canada; 2 Department of Botany & Biodiversity Research Centre, University of British Columbia, Vancouver, British Columbia, Canada; 3 Department of Zoology & Biodiversity Research Centre, University of British Columbia, Vancouver, British Columbia, Canada; 4 McCallum Environmental Consulting Ltd., Kamloops, British Columbia, Canada; 5 Department of Natural Resource Sciences, Thompson Rivers University, Kamloops, British Columbia, Canada; University of Saskatchewan, CANADA

## Abstract

Global analyses of bird communities along elevation gradients suggest that bird diversity on arid mountains is primarily limited by water availability, not temperature or altitude. However, the mechanism by which water availability, and subsequently primary productivity, increases bird diversity is still unclear. Here we evaluate two possible mechanisms from species-energy theory. The *more individuals hypothesis* proposes that a higher availability of resources increases the total number of individuals that can be supported, and therefore the greater number of species that will be sampled. By contrast, the *more specialization hypothesis* proposes that increasing resource availability will permit specialists to exploit otherwise rare resources, thus increasing total diversity. We used 5 years of surveys of grassland songbird communities along an elevational gradient in British Columbia, Canada, to distinguish between these hypotheses. Vegetation changed markedly in composition along the gradient and contrary to the expectations of the *more specialization hypothesis*, bird community composition was remarkably constant. However, both total abundance and species richness of birds increased with increasing water availability to plants. When we used rarefaction to correct species richness for differences in total abundance, much of the increase in bird diversity was lost, consistent with the expectations of the *more individuals hypothesis*. Furthermore, high species richness was associated with reductions in territory size of common bird species, rather than the fine-scale spatial partitioning of the landscape. This suggests that bird diversity increases when greater resource availability allows higher densities rather than greater habitat specialization. These results help explain a pervasive global pattern in bird diversity on arid mountains, and suggest that in such landscapes conservation of grassland birds is strongly linked to climate and hydrology.

## Introduction

Across North America, grassland songbirds have been declining in abundance for almost 40 years [1–3]. Recovery of these species will depend on maintaining and restoring high-quality breeding grounds. Presumably, increases in the availability of resources in an ecosystem such as water and nutrients lead to increases in the abundance of organisms at all trophic levels [4,5], and such increases would improve the quality of breeding grounds. However, there is still a strong debate about the mechanisms by which gradients in resource availability affect the number of species of either plants [6] or higher trophic levels such as songbirds [7–9].

In temperate grasslands, changes in the availability of abiotic resources such as water have been linked to changes in the richness, abundance, and reproductive success of many grassland bird species [10–14]. Effects of resources on birds is often thought to be mediated by vegetation change [15]. Certainly, plant characteristics such as live plant biomass, the mass or cover of plant detritus, and shrub abundance have often emerged as important predictors of grassland songbird abundance and species number [14]. However, when gradients in resource availability occur over large spatial scales (e.g. latitudinal gradients), the effect of resources can be confounded by other factors, such as the spatial turnover in species identity. Examining changes in grassland songbird communities along local plant productivity gradients, such as a single mountainside, provides an ideal opportunity to test the mechanisms of how grassland songbirds accumulate species with increases in water or nutrient availability to plants. Local gradients allow us to isolate these mechanisms while maintaining consistent species pools, disturbance patterns, and evolutionary histories [16,17]. Consequently, examining patterns of diversity on local gradients can provide insight into how diversity changes along broad latitudinal or continental-scale variation in water and nutrient abundance [8,18,19] and provide local tests of species-energy relationships.

Elevation gradients are particularly useful local gradients because they provide some of the most rapid spatial changes in abiotic resources. Along most elevation gradients, climatic variables have strong correlations with avian species richness and the abundance of individual birds [7,9]. However, although elevation gradients can circumvent some of the limitations of larger-scale environmental gradients, there are additional confounding factors to consider. Locations along elevation gradients become drier and colder with altitude [20] typically resulting in fewer species at higher elevation. In such cases, it is challenging to determine if a low number of species at high elevations is due to low plant productivity or the fact that montane species and their habitats are less widely distributed (i.e., geometric constraints). However, in some areas orographic precipitation produces an effect that results in dry warm valley bottoms (i.e., deserts) and wet cool mountain tops (i.e., grasslands), resulting in an increasing number of species at higher elevation. By using such an elevation gradient in this study, we were able to isolate the effects of resource availability from the otherwise confounding effects of elevation and geometric constraints. Indeed, recent global analyses of bird diversity over elevational gradients demonstrate distinctively different patterns between such arid mountains, where low elevations are driest, and humid mountains, where low elevations are wettest [9]. However, the mechanisms underlying these different patterns remain unresolved.

We examined the distribution of temperate grassland songbirds along an moisture gradient to determine if and how species number accumulates with plant productivity. We then tested two hypotheses that could explain how songbird diversity changes along this gradient. By examining how songbirds accumulate species along this gradient we provide insight into how these species will respond to changing climates and which management strategies could be used to recover declining populations.

**Fig 1:**
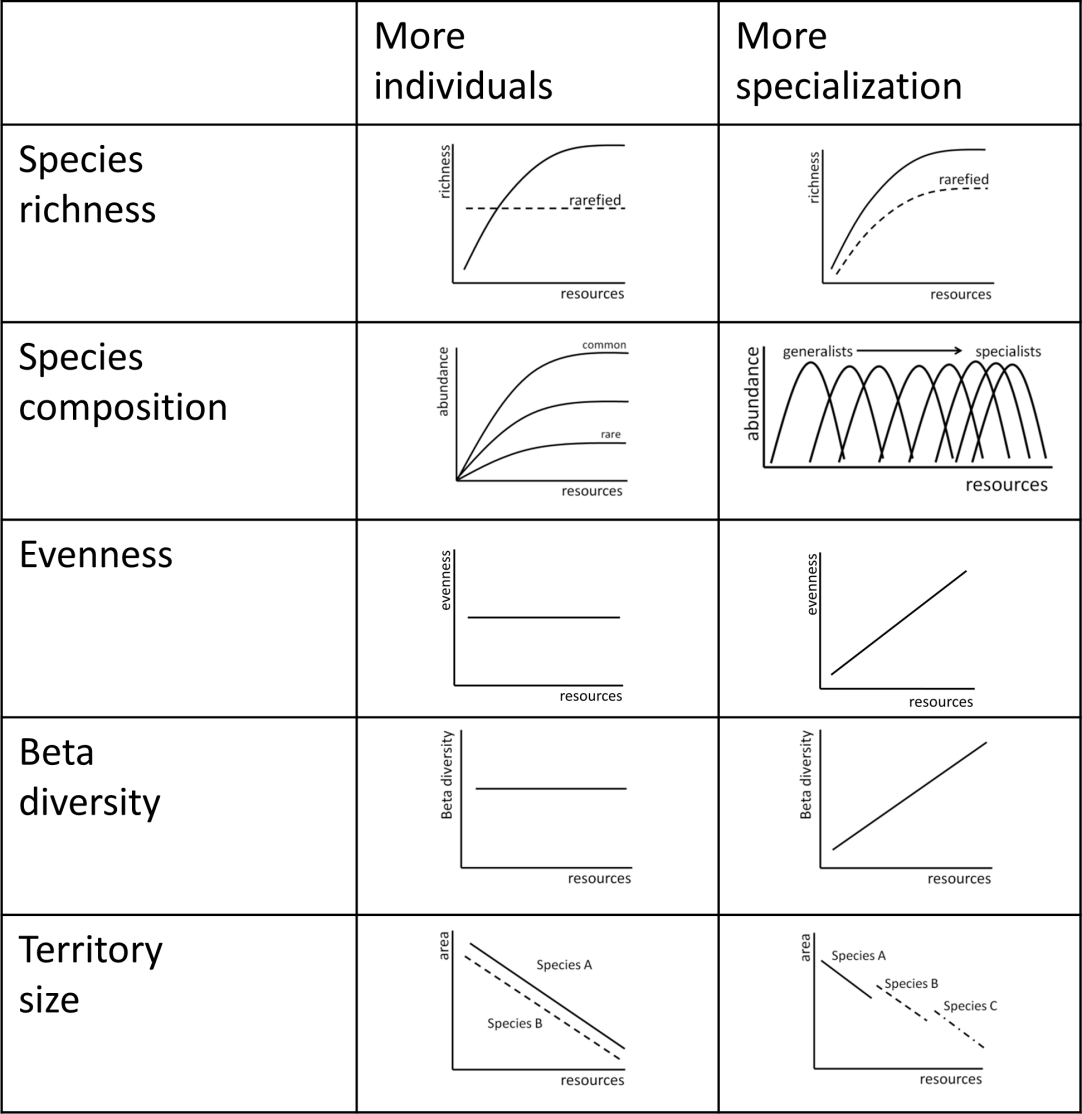
Predictions for mechanisms behind richness-productivity relationships in semi-arid and temperate grassland bird communities of south-central British Columbia, Canada. Each row represents predictions for how species richness, species composition, beta diversity, or territory size will change on a gradient of resources under either the *more individuals hypothese*s or the *more specialization hypotheses*.

Two alternative hypotheses propose mechanisms to account for changes in songbird abundance and species number under different levels of resource availability to plants [7,9]. Both hypotheses predict that as abiotic resources such as water increase, the number of songbird species will also rise. However, each hypothesis provides a different mechanism for the accumulation of species. Both provide a series of predictions for how the abundance of individuals, number of species, and structure of communities (identity and abundance of species) should change along a resource gradient (Fig 1). We use these predictions to provide evidence supporting or refuting each hypothesis.

The *more individuals hypothesis* asserts that the number of individuals increases linearly as a function of resource availability, and this increase in individuals is associated with more species through simple sampling effects [21]. Applied to our grassland birds, this hypothesis implies that an increase in water availability will increase with plant productivity and therefore the number of birds supported per unit area will also increase. Correcting for changes in abundance along the elevational gradient should therefore remove the increase in species diversity. In this case evenness of bird communities should remain unchanged because there are not close ties between species and niche [22]. Furthermore, because of the positive relationship between water availability and the number of songbird species is due solely to increased abundances rather than a change in the relative availability of niche, there will be little difference in the types or identity of grassland songbirds species along the gradient (“turnover”, [23]) adjacent locations will have similar species identity and relatives abundances (“variability”, [23]), and the relative distribution of individuals among species (“evenness”, [22]) should be constant over the gradient. The increase in bird abundance along the gradient may also be achieved through an accompanying decline in territory size in all species: at high resource availability, smaller areas are required to obtain sufficient energy.

Alternatively, the *more specialization hypothesis* predicts that one songbird species will replace another as resource availability changes [24,25]. When resources are scarce, only generalists or species that consume a broad range of resources can obtain sufficient resources to persist [21]. As water availability increases, rare resource types will become sufficiently abundant to support specialist songbird species. Consequently, increasing energy results in more species as a few generalist species consuming multiple resource types are replaced by multiple specialist species each consuming few resource types. In particular, the increase in niche at high productivity is expected to result in even distributions in abundance among species[22]. As a result, there should be a greater variation in songbird community structure within particular vegetation communities, especially in vegetation communities with low productivity. Assuming that resources are always heterogeneously distributed, then dry areas occupied by generalist species are expected to have lower beta diversity than wetter areas which are occupied by specialist species that show fine-scale spatial segregation.

To test if either of these hypotheses applies to grassland songbirds, we asked specific questions about the factors that structure grassland songbird communities at particular points along a local gradient of water availability to plants in south-central British Columbia, Canada. Firstly, does the number of individual grassland songbirds and number of songbird species change along the gradient? If we do detect patterns of change in the number of species and the number of individuals, are these changes associated with water availability? Second, what is the mechanism by which the number of species accumulates along the gradient? Are there changes in the number of species related to the abundance of songbird individuals, are there compositional changes in songbird communities (e.g., species turnover), and do territory sizes of individual species change along the gradient?

Each of the hypotheses we tested has different implications for conservation. If the number of species increases with water availability in a manner consistent with the more specialization hypothesis, grassland managers interested in promoting songbird species must provide for the specific habitat requirements of each individual species. If the number of species increases in a manner consistent with the more individuals hypothesis, grassland managers can provide general habitat requirements that benefit many species simultaneously. Thus, if species accumulate via the more individuals hypotheses, grassland managers interested in recovering songbirds can avoid intensive and expensive species specific management approaches and instead focus on practices that promote the broad-scale health of grasslands.

## Methods

We conducted songbird surveys and described vegetation characteristics in the Lac du Bois Provincial Park near Kamloops, British Columbia, Canada (50^o^39’59” N, 120^o^19’09” W). Mean monthly precipitation at the Kamloops weather station (345 m between 1981 and 2010 averaged 23.15 mm (range = 12.4 mm.an^-1^–37.4 mm.an^-1^). June and July have historically been the wettest months and February and March the driest. Mean daily temperatures over the same period averaged 9.2 ^o^C (range -2.8 ^o^C to 21.5 ^o^C). July and August are the hottest months and December and January the coldest [26]. Lac du Bois Provincial Park is a protected shortgrass and shrub-steppe ecosystem that occurs in the rain shadow of the British Columbia Coast Mountains. The entire region is characterized by a strong orographic effect. With increasing elevation, the dry valley vegetation transitions (at ~ 350 m) across a series of benches to wet, cool grasslands at the upper elevation forest boundary (at ~ 900 m). Plant communities along this moisture gradient vary widely (S1 Fig).

Local differences in precipitation, temperature, soil depth and texture, soil parental material, topography, and aspect contribute to the structure and composition of local plant and animal communities [27–29]. Dominant grasses include bluebunch wheatgrass (*Pseudoroegneria spicata* (Pursh) A.Love) and rough fescue (*Festuca scabrella* Rydb.). Common shrubs include big sagebrush (*Artemisia tridentata* Nutt.), rabbit brush (*Chrysothamnus nauseosa* (Pallas ex Pursh) Britton.), rose shrubs (*Rosa acicularis* Lindl.) and grey horsebrush (*Tetradymia canescens* Nutt.) [28]. The study area has been used extensively for livestock grazing, homesteading, and recreation for over 150 years [30], but currently it is primarily used for cattle grazing at low to moderate stocking rates [31]. Invasive and exotic species such as spotted knapweed (*Cenaurea maculaosa* Lam.), great mullein (*Verbascum thapsus* L.*)*, dalmatian toadflax (*Linaria dalmatica* L.), and cheatgrass are present but occur in low abundance. Canada bluegrass (*Poa compressa* L.) and Kentucky bluegrass (*Poa pratensis* L.) are perhaps the most common non-native plants but still occur only in low abundances.

To identify patterns of songbird diversity along the gradient, we counted grassland songbirds during the breeding season (mid-May to early July) using two methods: abundance surveys and territory mapping. Abundance surveys were conducted annually from 2008 to 2012 at 96 point locations over 5 years. Locations were grouped into six blocks, with 16 locations in each block (S2 Fig). Locations were separated by at least 250 m and the six blocks were distributed across and along the gradient. Abundance surveys consisted of visiting a location within 4 hours of sunrise and counting all grassland songbirds seen or heard within 100 m of the location during a 5-minute period. Each location was visited between three and five times during each breeding season. Abundances were determined by using the maximum number of individuals detected in a single count (i.e., singing males) and correcting this value using estimates of detectability [32]. To account for differences in detectability among species and sites we estimated detectability by year and species only for the five most common species using function gdistsamp in package unmarked v. 0.10–4 [33] in R 3.1.1 and specified the half normal key function. For these common species we used distance sampling to account for unequal detection of individual birds. For species with less than five records, we pooled records from all years and species and estimated detectability using function pcount with Poisson as the latent abundance distribution. The pcount function is part ofpackage unmarked v. 0.10–4 in R 3.1.1 [34]. For these rare species there was simply not enough data to reliably model detectability by distance. We used no specific predictors to account for changes in detectability between species or sites but instead used intercept only models as data in generalized linear mixed models (GLMMs) to relate abundance and species richness to site productivity. This allowed us to better account for correlation among sites.

To estimate territory sizes of individual species we surveyed 12 areas five times during the breeding season of 2008. Two mapping areas were associated with each of 6 blocks used for surveys. We ranked each of the 6 blocks based on amount of plant biomass from the 16 point count locations within that area. These are the same 6 blocks used as random effects in the mixed model analysis described below. Each mapping area consisted of four adjacent, 100 m wide transects, each 500 m long, for a total of 20 ha. Each survey started at sunrise and for 3 hours all visual or auditory observations of birds were recorded as the observer slowly traversed the 20 ha area. To delineate territories, we combined sightings for all five visits for individual birds, and then estimated the minimum convex polygon (MCP) for each individual songbird using the Minimum Bounding Geometry Tool in ArcGIS 10.2.2 for Desktop software [35].

Normalized Difference Vegetation Index (NDVI) correlates well with elevation and soil moisture measured at point locations along the gradient (see supplement). NDVI values were calculated from seamless Landsat Thematic Mapper Image take on 09 August 2007 and obtained from the British Columbia Government Geographic Data Warehouse. The grid cell size of these images was 30 m square.

To determine the distribution and abundance of plants, we surveyed plant functional groups (grasses, forbs, shrubs, trees) and the amount of bare ground at each of the 96 locations used in the songbird surveys. We estimated cover of each functional group using 20 m diameter circular plots, and measured the amount of living herbaceous plant material (live biomass) and dead herbaceous plant material (detritus mass) at each location using a destructive harvest of two 0.5 m x 0.5 m plots. We ranked survey blocks into cover classes from 1 (121.1 g.m^-2^) to 6 (326.3 g.m^-2^) based on the average amount of live biomass plus detritus mass collected at each location.

We summarized data at both the block (n = 6) and location (n = 96) scales. At each location we counted the number of individual singing males by species. Species richness was determined with a simple count of the number of species, and rarefied richness was calculated using function rarefy in package VEGAN in R 3.1.1 [36]. We rarefied to standardize species number along the entire gradient. We calculated two measures of α-diversity to account of the effect of abundance on species richness. To estimate songbird evenness, we used the Probability of Interspecific Encounter (PIE), as 1-D, where D = Simpson’s Diversity (*D = sum p_i^2)*. All calculations were performed using function Diversity in package VEGAN in R 3.1.1 [36]. We calculated and tested rarefied richness [37] using package VEGAN.

We tested for relationships between songbird species abundance, richness, rarefied richness, and evenness using generalized linear mixed models (GLMMs). Relative species abundance, species richness, rarefied species richness, or evenness were used as the response variables and NDVI, live plant biomass, detritus mass, or ground cover used as explanatory variables. We rarefied species richness by adjusting the number of species estimated at each location for abundance of individuals observed at the same location. We determined the most appropriate random effect structure by comparing the full model with different random effect structures using Akaike Information Criteria (AIC). The most parsimonious random effects structure allows only the intercept to vary between both blocks and years. Analyses were done using the function lmer or function glmer in package lme4 [38] in R 3.1.1. We specified Gaussian errors in GLMMs for abundance and evenness relationships because our corrected estimates of abundance were no longer integers, and Poisson errors for richness relationships because they were counts. The strength of these relationships was assessed by examining whether the slopes of the regression lines were different than zero or different than other treatments. We assessed the existence of a hump-shaped relationship with abundance, richness or evenness by testing if a quadratic form of the statistical model fit the data better than the simpler linear form. Where appropriate we used pseudo-R^2^ values to describe the model fit by both fixed and random factors (i.e., the conditional R^2^).

To test for changes in community composition and species composition along the gradient, we calculated *β*-diversity in two ways following [23]. First, we tested for turnover in songbird communities along the gradient of either live biomass or detritus mass by first calculating a Bray-Curtis distance matrix to estimate the distance between songbird communities (Sorensen’s Dissimilarity) at each of the 96 points as well as another distance matrix measuring dissimilarity as the difference in pair-wise comparisons of the amount of live biomass or detritus mass (T3, T4 in [23]). These two matrices were compared using Mantel tests and generalized linear mixed models. Detrended Correspondence Analysis (DCA) was applied to the pair-wise matrix of live biomass and detritus mass to remove any bias the distance matrix calculations may have imposed on the linear nature of the gradient. We did not perform a similar analysis with shrub cover because a large number of locations had no shrubs and fitting a simple statistical model was not possible.

As a second way of calculating a more local estimate of beta diversity, we tested for a consistent change in the variability in community composition within blocks along the gradient (V4 in [23]). We ranked blocks based on their mean NDVI (n = 16/block), and then calculated how different the community composition of songbirds at each point was from the group mean (i.e., dispersion within blocks). The function BETADISP in package VEGAN was used to calculate the amount of dispersion in songbird communities from the typical community (the centroid) of that block. By comparing values at each of the six blocks ranked in order of mean biomass, we estimated variability of songbird communities within blocks. We fit generalized linear models (GLMs) or GLMMs to test for the relationship between vegetation descriptors and variability in songbird community composition within blocks. We assessed the nature of this relationship by examining the parameters in the statistical models we had fit.

## Results

**Fig 2:**
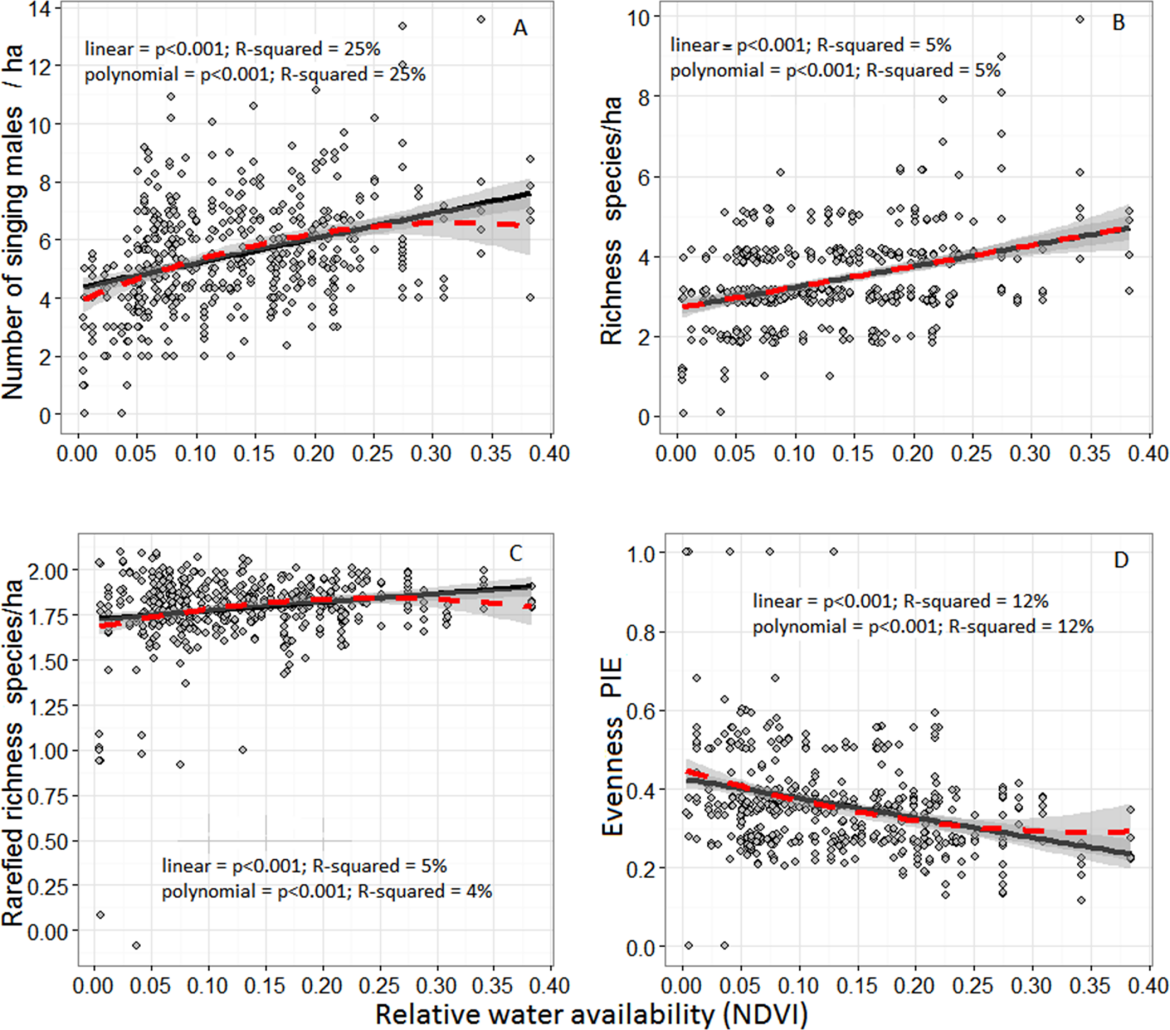
**Avian abundance as the number of singing males (A), richness (B), rarefied richness (C), and species evenness (PIE) along a gradient of relative water availability to plants (NDVI).** Songbird communities were surveyed during the breeding season from 2008–2012 at 96 locations. Solid lines represent the fitted values of generalized linear models with a single term, and dotted red lines represent a similar fitted but polynomial (curved) model. Generalized linear mixed models were used to test for goodness of fit of models to data and whether straight or curved lines fit the data better. In all cases the straight line model fit (solid black lines) the data better than the curved ones (dotted red lines). Shaded areas represent 95% confidence intervals.

**Fig 3:**
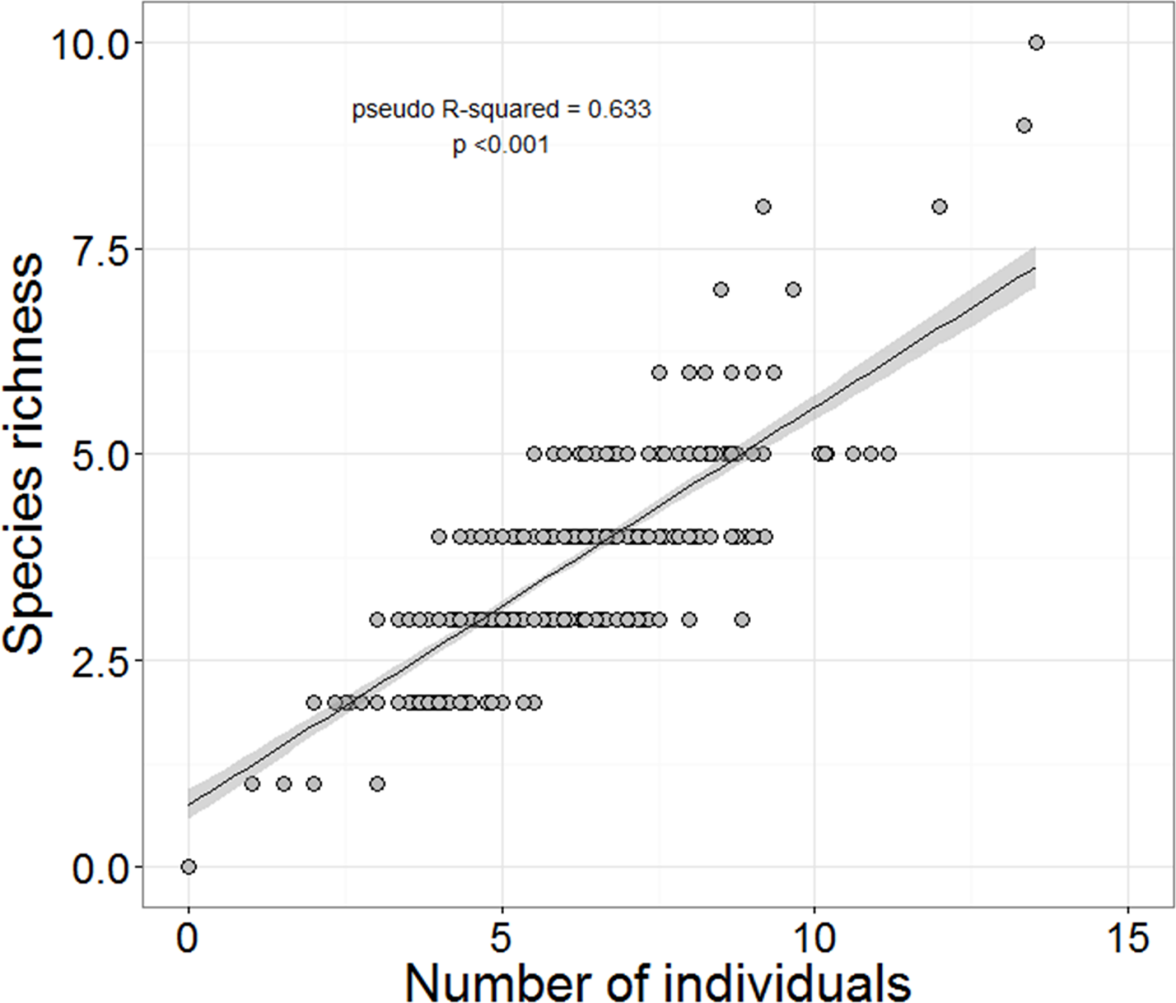
Relationship between the richness of grassland songbird species and the abundance of grassland songbirds. Each data point represents the estimated density and number of songbird species at a single location in one year. The estimated relationship is shown with shading representing the 95% confidence interval. For simplicity, we do not show error in estimating the number of individuals or species in this figure.

**Fig 4:**
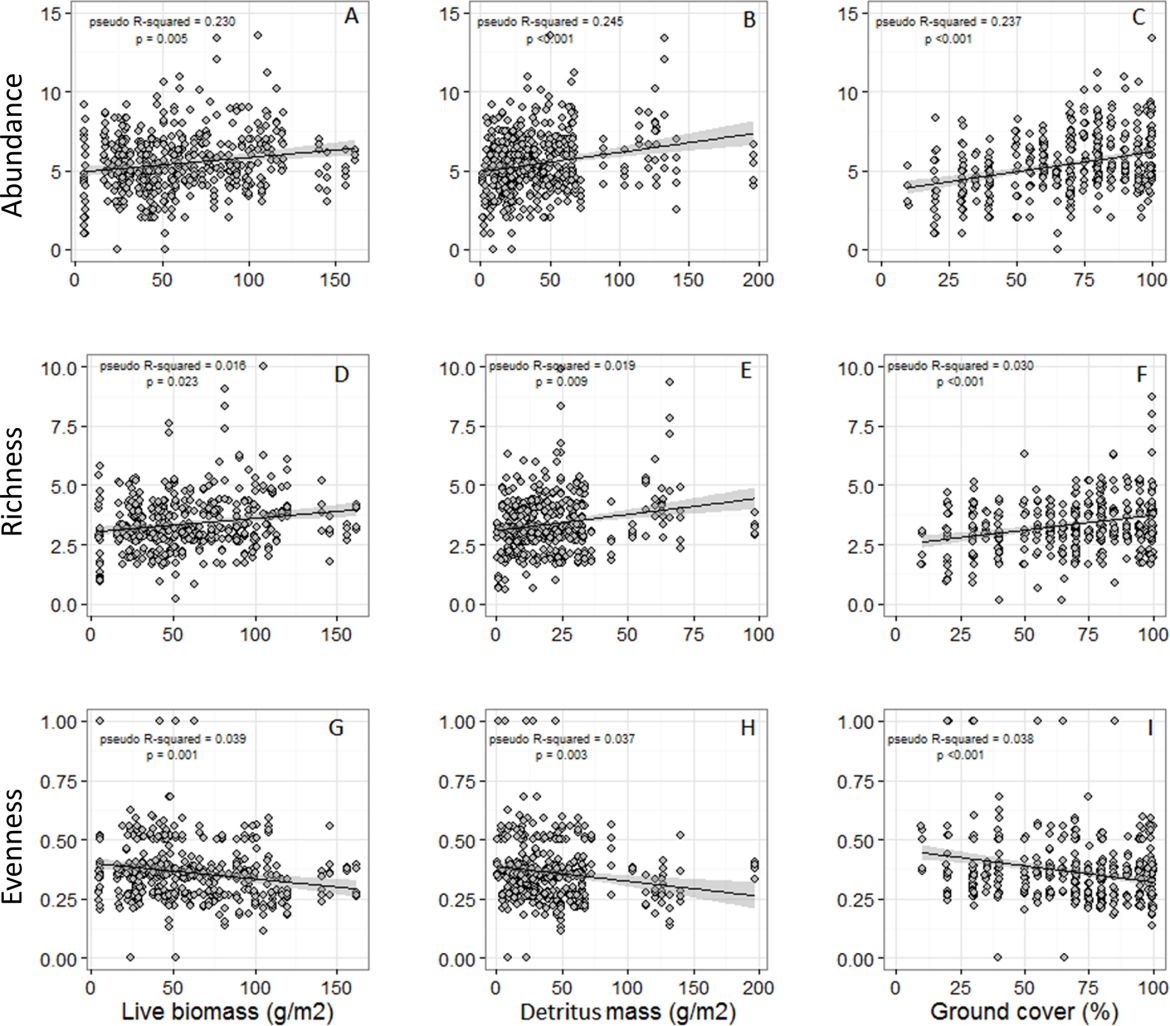
Relationship between abundance, number of species (richness), and species evenness (PIE) with live biomass, detritus mass, and ground cover. Points indicate observations of songbird communities at 96 locations made from 2008–2012. Lines are linear model fits with shaded 95% confidence intervals.

The abundance, richness, and evenness of grassland songbird communities changed along the gradient of water availability to plants (Fig 2). As elevation increased, so did bird abundance and species richness, although this relationship was unimodal with peak individual abundance at 783 m and a corresponding peak in the number of species at 783 m. Our proxy of water availability and net primary productivity (NDVI) increases as a curvilinear function of elevation (R^2^ = 59%, quadratic regression F_4, 6_ = 27.3, p <0.001, S3 Fig), so abundance and species richness also increased with NDVI. Additionally, the number of species increased linearly with the abundance of individuals (Fig 3, S1 Table). However, the linear relationships were as good a descriptor of the pattern (difference between linear and polynomial models: abundance χ^2^ = 0.01, p = 0.916, richness χ^2^ = 0.11, p = 0.734). There was no relationship between the abundance of birds and shrubs (χ^2^_4, 5_ = 0.49, p = 0.480), but bird abundance did increase with increasing ground cover (χ^2^_4, 5_ = 12.1, p <0.001; Fig 4C), live plant biomass (χ^2^_4, 5_ = 7.78, p = 0.005), and detritus mass (χ^2^_4, 5_ = 17.05, p < 0.001; Fig 4B). Species richness similarly was unrelated to shrub cover (χ^2^_3, 4_ = 2.13, p = 0.150), but increased with ground cover (χ^2^_3, 4_ = 11.9, p < 0.001; Fig 4F), live plant biomass (χ^2^_3, 4_ = 5.0, p = 0.026; Fig 4D), and detritus mass (χ^2^_3, 4_ = 6.57, p = 0.010; Fig 4E). All told, both live plant and detritus biomass provide the best predictors of songbird abundance and richness along the gradient and this relationship is roughly linear.

Along the gradient, the number of individuals increased more quickly than the number of species (Fig 3). While rarefied species richness still significantly increased with NDVI, rarefaction greatly diminished the magnitude of the NDVI effect (Fig 2C). Rarefaction removed the significant association of species number with plant live biomass (Fig 4). Thus, in general when we account for the increase in abundances the number of species per location (rarefied richness) remains relatively constant along the gradient.

Mantel tests did not show any association between changes in songbird communities and resource availability (Table 1). Likewise, GLMMs showed no change in species identity along the gradient of either live biomass (F_4, 5_ = 0.42, p = 0.52) or detritus mass (F_4, 5_ = 0.01, p = 0.91). There was also no strong spatial variability in songbird communities along the gradient when we ranked blocks based on either live biomass, detritus mass, or bare ground (F_5,90_ = 1.51, p = 0.194). Finally, although evenness did decrease with NDVI (χ^2^_4, 5_ = 31.9, p < 0.001), live biomass (χ^2^_4, 6_ = 13.88, p < 0.001) and detritus mass (χ^2^_4,5_ = 8.61, p = 0.003), this relationship was opposite to the increase in evenness predicted by the more specialization hypothesis. Songbird communities did not change in composition or increase in their variability along the gradient. More individuals of the same species were present as the amount of live plant or detritus mass increased along the gradient. Likewise, community composition changed little between adjacent locations. If anything, dominant species became slightly more dominant, and evenness was reduced with increasing water availability and plant production.

**Table 1:** Results of Mantel tests examining the relationship between change in community composition of songbirds between sites on the gradient and change in either live biomass or detritus mass between sites for each of five years in which we did surveys. Values represent the correlation, either negative or positive, between community change and environmental change. Values in parentheses are the p-value from significance tests.

Year	Live Biomass	Detritus mass
**2008**	-0.08 (0.921)	0.07 (0.122)
**2009**	-0.09 (0.934)	0.10 (0.055)
**2010**	-0.03 (0.714)	-0.04 (0.726)
**2011**	-0.15 (0.997)	0.07 (0.081)
**2012**	-0.10 (0.944)	0.14 (0.017)

**Fig 5:**
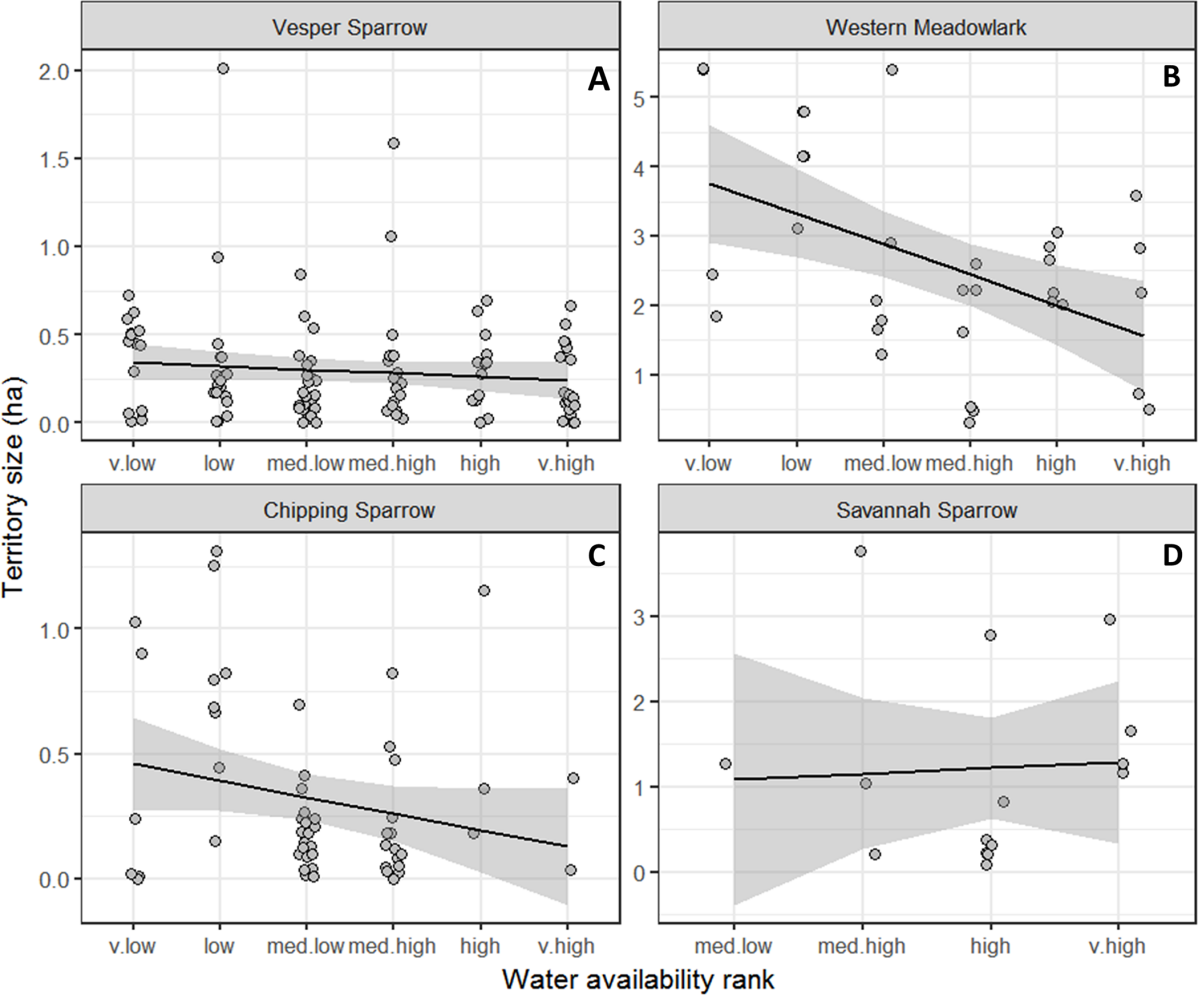
Territory size estimates for the four most common species of grassland birds observed during the breeding season of 2008. Observations were made over a range of grassland types. Each point represents the size of an individual territory measured at one of two 20 ha sites in ranked categories of water availability. Ranks run from where water is very low (v.low) to where water is abundant very high (v.high). Points are offset over productivity ranks to show overlapping points. Relationships are shown as generalized additive models with shading representing 95% confidence intervals.

We estimated the territory size of 203 individual birds from four common species along the gradient. Vesper Sparrows (n = 102), Western Meadowlarks (n = 31), Chipping Sparrows (n = 55) and Savannah Sparrows (n = 15) each had three or more sightings over the five days of survey at each location. The size of Vesper Sparrow (F_5_ = 0.93, p = 0.464) and Savannah Sparrow (F_5_ = 0.96, p = 0.444) territories did not change along the gradient. However, territory sizes for Western Meadowlark (F_5_ = 4.31, p = 0.005) and Chipping Sparrow (F_5_ = 5.79, p <0.001) were lower in areas where water was abundant than in areas where water was scarce. Territory size for individual species did not change with the amount of live biomass, detritus mass, shrub or bare ground cover. Generally, territory size declines with increased live plant biomass, detritus mass, and increases with the amount of bare ground, but this relationship is highly variable (Fig 5).

## Discussion

We show that songbird species accumulate along a gradient of water availability to plants consistent with the more individuals hypothesis. Our data show that there is a linear and generally consistent increase in both the number of individuals and number of species of grassland songbirds with increasing water availability to plants. This occurred because common birds either had smaller territories or greater territory overlap at high water availability. It did not occur because resource generalists were supplanted by specialists. Specifically, the identity of the dominant species in songbird communities did not change along the gradient, nor did beta diversity along the gradient increase with water availability. Furthermore, when we account for change in abundance over the elevation using rarefaction, the diversity pattern disappears. Species evenness decreased as water availability increased, opposite to the expectations of the more specialization hypothesis. Taken together, these data provide much stronger support for the more individuals hypothesis than for the more specialization hypothesis, and suggest that songbird species in temperate grasslands might accumulate diversity on gradients of abiotic resources in ways different than songbirds in other biomes. Semi-arid ecosystems experience periodic resource abundance interspersed with long periods of resource shortages or drought [39–42]. When resources are in short supply or resource availability is periodic, consumers must be opportunistic when choosing prey especially in time of resource shortage [43]. This makes specialization difficult for species occupying ecosystems at the lower end of their resource tolerance. At a broader scale, specialization of species in tropical communities maybe greater than that observed here due to the relative stability of climate in tropical versus temperate ecosystems. These results are important for two reasons. First, by providing contrasting results to more humid tropical systems [7,15,44–46], we demonstrate that the mechanisms driving species accumulation along resource gradients may be dependent on the patterns of resource abundance and shortage in the ecosystem. Our finding of a unimodal peak in bird diversity are in agreement with a global analysis of arid mountains [9]. Second, we examined not only the shape of the richness-productivity relationship in upper trophic levels, but provided a concrete approach to test why these patterns occur.

We suggest that despite large changes in both water availability and plant community structure along the gradient (S1 Fig), songbird species accumulate along gradients in temperate grasslands by first adding new species at low abundances. New rare species were added to songbird communities incrementally as water availability to plants increased, and these species generally remained rare as water availability increased. Thus, the richness-productivity relationship in grassland songbirds relies on the addition of less common species through simple probability. When abiotic resources such as water increase, plant growth increases and there is a corresponding increase in the number of songbird individuals because more plant growth can support an abundance of arthropods on which songbirds forage. As the number of songbird individuals supported at a particular location increases, it is likely that some of these species will be not be the same as the species already present. Thus, species richness increases. In areas where water is scarce, very few individuals occur and either sampling or priority effects favor species that are more common in the regional species pool.

One key pattern supporting the more individuals hypothesis is that the same two songbird species (Western Meadowlark and Vesper Sparrow) remained dominant along the entire gradient. Although some rarer species such as the Brewer's Sparrow or Savannah Sparrow do not occur along the whole gradient we studied, other rare species such as Western and Mountain Bluebirds and American Goldfinch have a broad distribution on the gradient we studied. Thus, we conclude that similar to studies in other grassland bird communities [47] there is no strong specialization of birds with respect to particular resources in the temperate grasslands we examined, and that the number of species increases primarily as a function of ecosystem-level productivity. This is in contrast to other bird communities [15,44–46] and other vertebrate species [48] which typically show strong changes in species identity along gradients.

The mechanisms that regulate avian diversity along resource gradients may depend on the nature of the gradient. We speculate that dry ecosystems, with variable or uncertain resource availability, may accumulate diversity in a manner consistent with the more individuals hypothesis. This may occur because interspecific competition for specific resources is limited along the gradient [12,49,50]. Strong competition for resources at this local scale would be unlikely in ecosystems with periodic or uncertain resource availability [43,51]. Conversely, wetter ecosystems that are less prone to resource shortage accumulate diversity in a manner consistent with the more specialization hypothesis.

The distribution of grassland songbirds we observed does not match the change in species identity and increasing species richness with plant productivity typically described in tropical songbirds [7,15,44,46], but the patterns of changing species identity and species richness with plant productivity we observed do match results from dry grassland communities [12,52]. In tropical forests there are often large changes in species identity along the gradient linked to marked changes in vegetation structure [7,15,46]. Additionally, tropical humid gradients often demonstrate declining plant productivity with elevation which places geometric constraints on the number of species. Since the temperate arid ecosystem we examined has the reverse trend in resource availability to humid gradients, it may be that the patterns of changing species identity on gradients seen in tropical humid systems arises from geometric constraints on species number placed by decreasing productivity at higher elevations. The amount of physical space that species require may result in changes in species identity on gradients. A different distribution of resources resulting from different topographies could restrict species that have large patch size requirements. Removing these geometric constraints could allow the same species to exist, b by allowing these species to simply change the area over which they forage. For example, in eastern Oregon, the number of species of shortgrass prairie and shrub-steppe songbirds was also found to be dependent on the abundance of peripheral or wide-ranging species rather than resident species that dominate local communities [53], and in our study we observed declining territory sizes in the dominant songbirds with increased plant productivity.

Another reason our results may differ from those in tropical ecosystems other than geometric constraints is that scarce and periodically available resources could change species traits in semi-arid ecosystems. Interspecific competition for specific resources by grassland birds is limited in similar semi-arid ecosystems [12,49,50], and our results are also consistent with this more detailed habitat and behavioral work on songbird communities. Although few studies relate abundance or community structure of songbirds to particular vegetation characteristics [54], songbird abundance in these semi-arid systems is generally positively associated with prey abundance [47] or increased vegetation structure [55,56]. In studies covering larger areas across the Great Basin of the western United States, songbird species are associated with the density of shrubs and woody vegetation or more open vegetation [57]. However, despite these associations, grassland songbirds consistently fail to show large differences in species composition between habitat types [12,58]. This is again consistent with the more individuals hypothesis being better supported in dry ecosystems. Future studies should focus on isolating the effects of geographic constraints and the availability or periodicity of resource availability to plants in determining patterns of species accumulation in songbirds.

Our findings are important not only to aid in our understanding of the factors that determine community structure in grassland songbirds, but also because they inform conservation efforts and management actions [57,59]. Both the number of species and the abundance of individual songbirds in temperate grasslands could be increased by promoting processes that lead to greater plant productivity. Plant productivity can be increased by allowing detritus to accumulate. Detritus accumulation can be promoted by reducing the frequency of disturbances such as prescribed fire and livestock grazing. This would be especially true in areas with low to moderate water availability or areas where climate change in increasing the frequency or severity of drought. Tall grass prairie studies have found that the accumulation of songbirds peaks at 3 to 4 years following disturbances such as fire or other disturbance [60–63]. It may be that increasing grazing intensity but extending periods of no grazing in grasslands such as the ones we study could allow the recovery of both plant species and higher trophic levels through the accumulation of detritus without reducing livestock production.

Our study was performed in only a single location that was quite far north. Some common grassland species such as grasshopper sparrow (*Ammodromus savannarum*) or lark sparrow (*Chondestes grammacus*) are rare or absent from grasslands such as these and thus further studies, preferably with grazing trials that vary detritus amount should be done to assess the widespread applicability of our results. Following from the more individuals hypothesis, increased detritus may allow upper trophic levels, such as arthropods and songbirds, to reach higher levels of abundance and promote the accumulation of species. At drier locations, during periodic drought, or to mitigate climate change effects, grassland managers could promote the accumulation of detritus to increase the number of songbird species. In our study area, dry grasslands are expected to become more widespread and wetter grasslands could disappear. This could reduce habitat and thus reduce both the abundance of songbirds and the number of species present. Thus, experiments manipulating grazing frequency and intensity on rotations of 4 to 5 years are essential in determining not only how rest-rotation grazing systems influence plants but also how they can promote songbirds.

## Conclusion

These findings are important because they point to different mechanisms that underlie general increases in the number of songbird species with ecosystem productivity that are dependent on resources availability. Semi-arid and temperate grasslands such as the one we studied often have periodic resource shortages that can dictate characteristics of species accumulation at both the plant and higher trophic levels [39,41,42,64]. We suggest that persistent resource shortage can lower plant productivity and reduce the availability of resources to species in upper trophic levels. In the grasslands we examined, this reduces the number of songbird species. Wetter ecosystems such as tropical forest, have more consistent patterns of resource abundance and in these ecosystems species could accumulate through different mechanisms than in the semi-arid ecosystems we studied. Determining the mechanism through which diversity accumulates is important because knowing the mechanism can provide both a prediction for how changes in diversity will occur when the climate changes, but also provide guidance for how to manage ecosystems to promote diversity. If diversity accumulates through processes such as those described by the more individuals hypothesis, we may be able to manage many species simultaneously and thus reduce management costs and efforts. In semi-arid and temperate grasslands, managing with ecosystem-wide techniques that promote plant productivity may also allow managers to mitigate the effects of climate change by proactively improving habitat quality for a broad range of species.

## Supporting information

S1 Table List and description of species encountered. (PDF)

S1 Fig Broad vegetation types from along the gradient. (TIF)

S2 Fig Study area map. (TIF)

S3 Fig Relationship between elevation and NDVI and elevation and soil moisture. (TIF)
