# Supplementary material for: Temperate grassland songbird species accumulate incrementally along a gradient of primary productivity
Source: PLoS One. 2017 Oct 23;12(10):e0186809. doi: 10.1371/journal.pone.0186809 (PMC5653332; doi:10.1371/journal.pone.0186809)
Supplement: S1 Table — (PDF) [file pone.0186809.s001.pdf]

S1 Table. List of species encountered during point count surveys at 96 locations along a moisture gradient near Kamloops BC, Canada between 2008 and 2012. Life history descriptions for habitat, food, nesting and foraging behaviour descriptions are those outlined published by The Cornel Lab of Ornithology (www.allaboutbirds.org) species guide.

**Common Name**	**Scientific Name**	**General habitat**	**Primary food**	**Nesting behaviour**	**Foraging behaviour**
American goldfinch	*Spinus tristis*	Open woodland	Seeds	Shrub	Foliage gleaner
American robin	*Turdus migratorius*	Open woodland	Insects	Tree	Ground forager
Black-billed magpie	*Pica hudsonia*	Open woodland	Omnivore	Tree	Ground forager
Brown-headed cowbird	*Molothrus ater*	Grassland	Seeds	Tree	Ground forager
Brewer's sparrow	*Spizella breweri*	Scrub	Insects	Shrub	Foliage gleaner
Clay-coloured sparrow	*Spizella pallida*	Scrub	Seeds	Shrub	Foliage gleaner
Cedar waxwing	*Bombycilla cedrorum*	Open woodland	Fruit	Tree	Foliage gleaner
Chipping sparrow	*Spizella passerina*	Open woodland	Seeds	Shrub	Ground forager
Common yellowthroat	*Geothlypis trichas*	Scrub	Insects	Shrub	Foliage gleaner
Dark-eyed junco	*Junco hyemalis*	Forest	Seeds	Ground	Ground forager
Eastern kingbird	*Tyrannus tyrannus*	Grassland	Insects	Tree	Flycatching
Flycatchers (Dusky/Alder)	*Empidonax oberholseri/E. alnorum*	Open woodland/Scrub	Insects	Scrub	Flycatching
Golden-crowned kinglet	*Regulus satrapa*	Forest	Insects	Tree	Foliage gleaner
Mountain bluebird	*Sialia currucoides*	Open woodland	Insects	Cavity	Flycatching
Mountain chickadee	*Poecile gambeli*	Forest	Insects	Cavity	Foliage gleaner
Nashville warbler	*Oreothlypis ruficapilla*	Forest	Insects	Ground	Foliage gleaner
Orange-crowned warbler	*Oreothlypis celata*	Forest	Insects	Ground	Foliage gleaner
Olive-sided flycatcher	*Contopus cooperi*	Open woodland	Insects	Tree	Flycatching

Pine siskin	*Spinus pinus*	Open woodland	Seeds	Tree	Foliage gleaner
Pygmy nuthatch	*Sitta pygmaea*	Forest	Insects	Cavity	Bark forager
Red-breasted nuthatch	*Sitta canadensis*	Forest	Insects	Cavity	Bark forager
Rock wren	*Salpinctes obsoletus*	Mountains	Insects	Ground	Ground forager
Rusty blackbird	*Euphagus carolinus*	Forest	Insects	Tree	Ground forager
Savannah sparrow	*Passerculus sandwichensis*	Grassland	Insects	Ground	Ground forager
Song sparrow	*Melospiza melodia*	Open woodland	Insects	Shrub	Ground forager
Spotted towhee	*Pipilo maculatus*	Scrub	Omnivore	Ground	Ground forager
Tree swallow	*Tachycineta bicolor*	Lake/Pond	Insects	Cavity	Aerial forager
Vesper sparrow	*Poosecetes gramineus*	Grassland	Insects	Ground	Ground forager
Violet-green swallow	*Tachycineta thalassina*	Open woodland	Insects	Cavity	Aerial forager
Warbling vireo	*Vireo gilvus*	Open woodland	Insects	Tree	Foliage gleaner
Western bluebird	*Sialia mexicana*	Open woodland	Insects	Cavity	Flycatching
Western kingbird	*Tyrannus verticalis*	Grassland	Insects	Tree	Flycatching
Western meadowlark	*Sturnella neglecta*	Grassland	Insects	Ground	Ground forager
Western tanager	*Piranaga ludoviciana*	Forest	Insects	Tree	Foliage gleaner
Wilson's warbler	*Cardellina pusilla*	Scrub	Insects	Ground	Foliage gleaner
Western wood-pewee	*Contopus sordidulus*	Open woodland	Insects	Tree	Flycatching
Yellow-rumped warbler	*Setophaga coronata*	Forest	Insects	Tree	Foliage gleaner
