# Supplementary figures and images for: Temperate grassland songbird species accumulate incrementally along a gradient of primary productivity
Source: PLoS One. 2017 Oct 23;12(10):e0186809. doi: 10.1371/journal.pone.0186809 (PMC5653332; doi:10.1371/journal.pone.0186809)

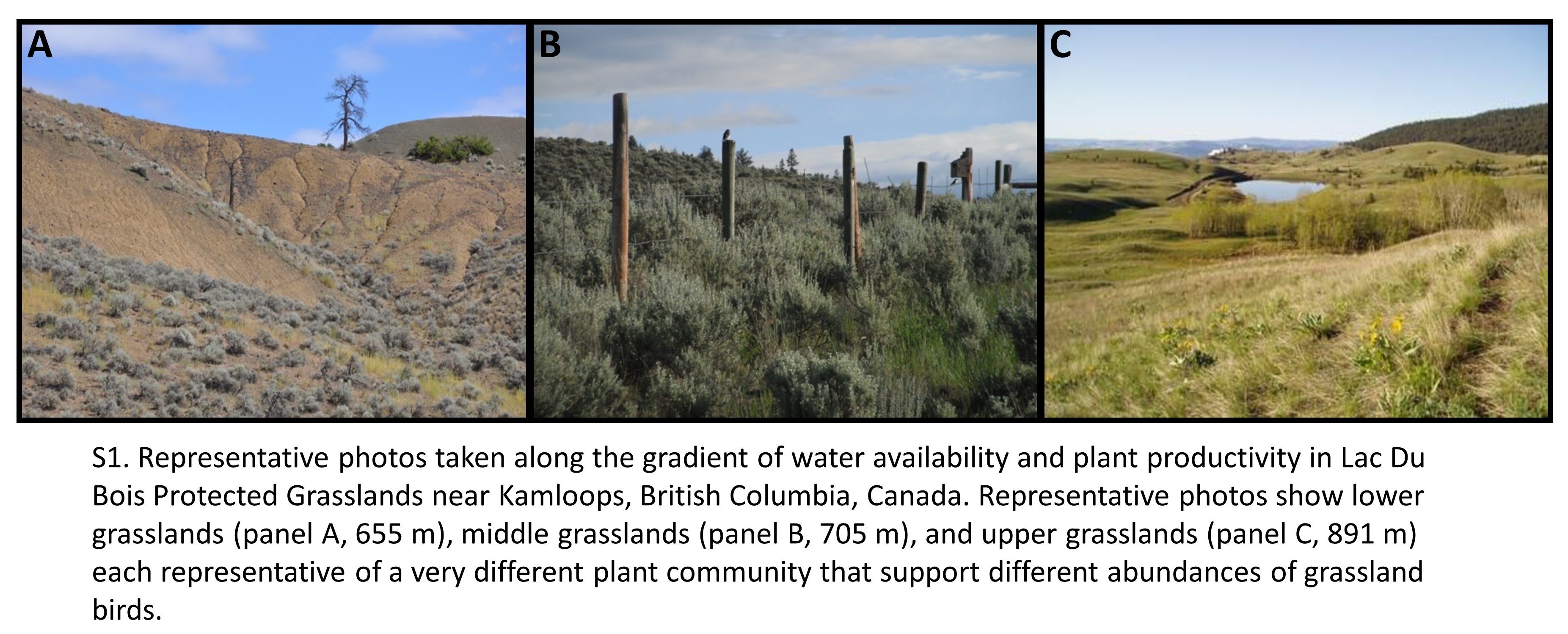

Supplement: S1 Fig — (TIF) [file pone.0186809.s002.tif]

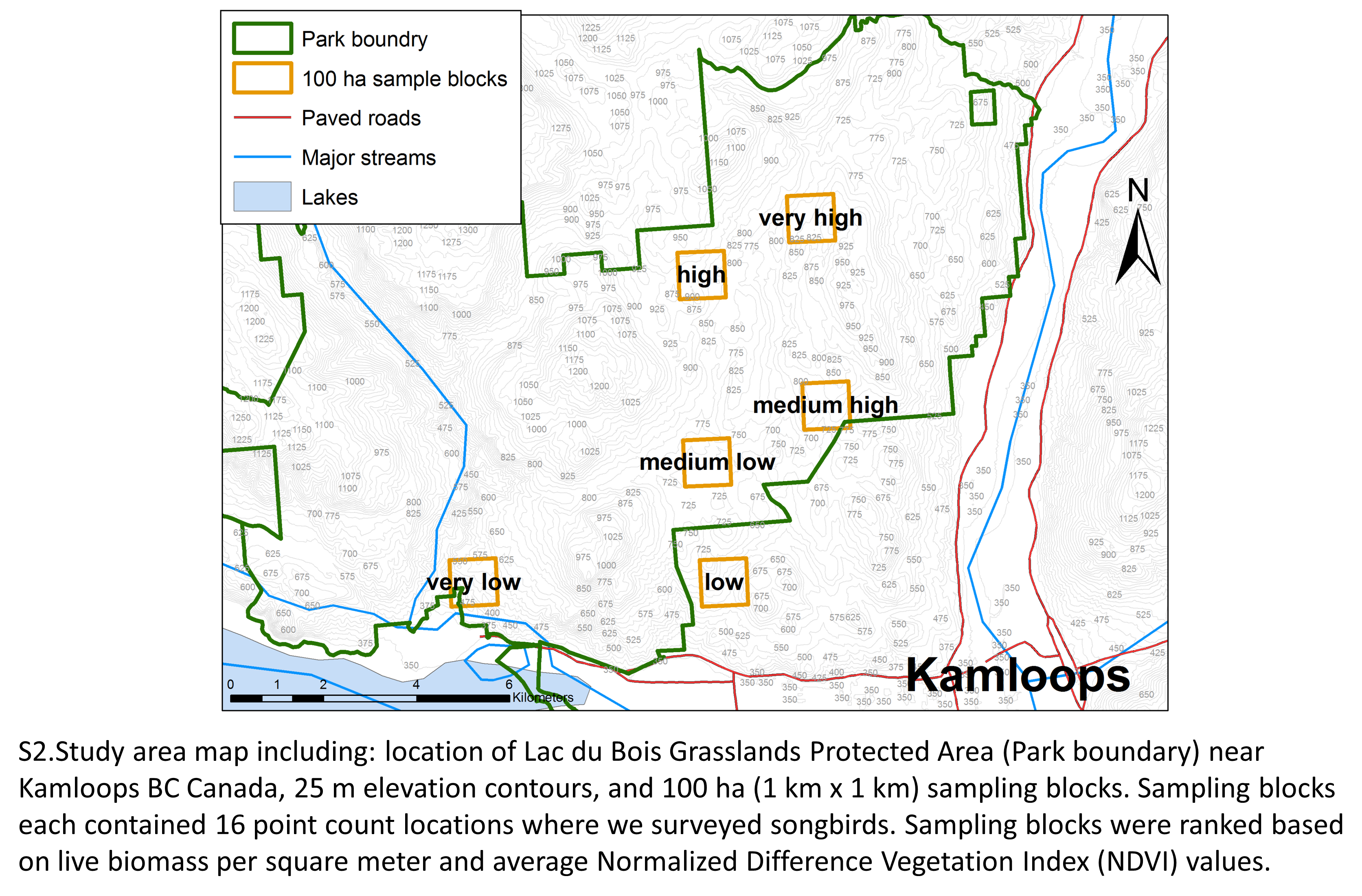

Supplement: S2 Fig — (TIF) [file pone.0186809.s003.tif]

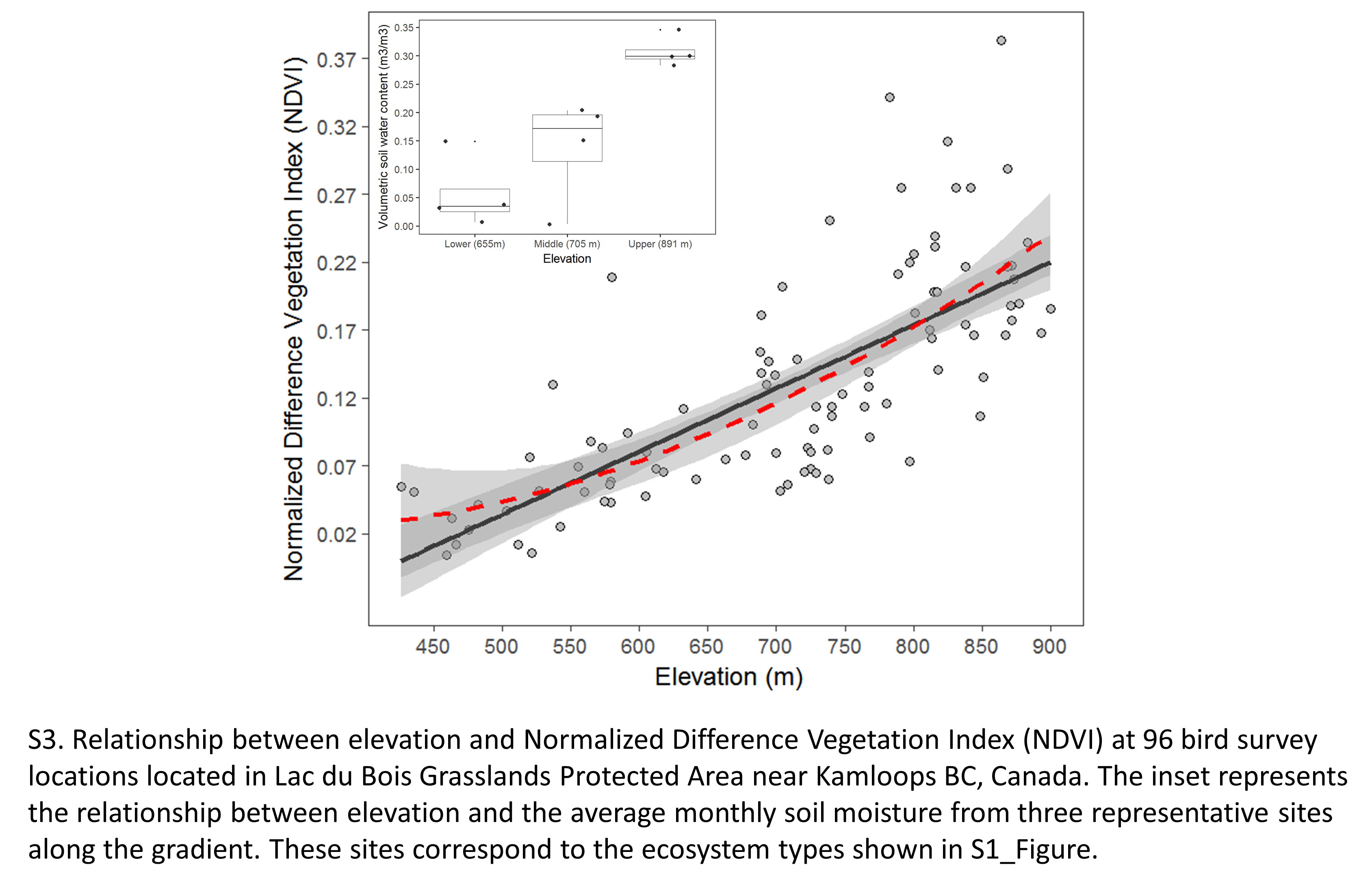

Supplement: S3 Fig — (TIF) [file pone.0186809.s004.tif]
